# The utilization of mangosteen pericarp extract for anticoccidial drug replacement in broiler feed

**DOI:** 10.1080/23144599.2022.2128271

**Published:** 2022-10-12

**Authors:** Pichet Sriboonyong, Pattaraporn Poommarin, Janjira Sittiya, Praneet Opanasopit, Tanasait Ngawhirunpat, Prasopchai Patrojanasophon, Chaiyakarn Pornpitchanarong

**Affiliations:** a Faculty of Animal Sciences and Agricultural Technology, Silpakorn University, Petchaburi IT Campus, Thailand; b Pharmaceutical Development of Green Innovations Group (PDGIG), Faculty of Pharmacy, Silpakorn University, Nakhon Pathom, Thailand

**Keywords:** α-mangostin, broilers, mangosteen pericarp extract, anticoccidial drug

## Abstract

The use of anticoccidial drugs in broilers has led to concerns, especially the drug residues in meat and the occurrence of drug resistance. This study aimed to extract, standardize, quantify and utilize mangosteen pericarp extract (MPE) containing α-mangostin as a replacement for anticoccidial drugs in broiler feed. The pericarp was acquired from different areas of Thailand and used for extraction and standardization. The antioxidant activity of the extract was evaluated. The extract was formulated into granules, and the flowability and stability of the granules were assessed. The MPE formulation was added to the broiler feed and then fed to the broilers that were infected with *Eimeria tenella*. The growth rate and intestinal lesion score (post-mortem) of the broilers were assessed. The pericarp obtained passed the identification test and phytochemical analyses. The active compound, α-mangostin, was best extracted using 95% ethanol. The MPE had superior antioxidant activity compared to standard antioxidants. Granules of the extract formulated with Avicel® PH102 provided desirable flowability and stability. The broilers fed with the feed containing 500 mg/kg α-mangostin showed a similar growth rate and post-mortem lesion score compared with the control group and those that received feed containing 60 mg/kg salinomycin. Our findings demonstrated that MPE with a high content of the active compound could be developed and used in place of anticoccidial drugs in the broiler feed.

## 1. Introduction

Broilers refer to chicken explicitly bred for meat production. They play a significant role in poultry husbandry, the food industry and the economy [1,2]. One of the major problems in poultry breeding is coccidiosis caused by a parasitic infection. The infection of protozoa from the genus Eimeria causes diseases in cattle, sheep, goats, buffaloes, rabbits and poultry. For broilers, the infection is found to reduce chicken growth, feed conversion and meat production performance, as well as other health concerns [3–5]. The infection causes metabolism malfunction, nutrient malabsorption (*E. acervulina* and *E. mitis*), intestinal inflammation (*E. brunetti* and *E. maxima*), villar destruction and haemorrhage due to intestinal damage related to *Eimeria* infection (*E. necatrix* and *E. tenella*). The infection severity is usually assessed post-mortem as an intestinal lesion score [6,7].

Antibiotics are used in poultry feed and water to prevent and treat coccidia infection resulting from sporulated oocyst ingestion. Different anticoccidial drugs are used nowadays, including amprolium, quinolines, folic acid antagonists (sulphonamides, 2,4-diaminopyrimidines and ethopabate), ionophores (monensin, salinomycin, lasalocid, narasin, maduramicin and semduramicin) and so on. Among the above-mentioned, ionophores were found to be the most important class of anticoccidial drugs; however, drug resistance has emerged and is widespread in broilers, limiting the drug efficacy. Moreover, it has been reported that overuse of some antibiotics can also decrease feed consumption of chickens, and some anticoccidial agents were found as residues in the meat [8,9]. Thus, the replacement of anticoccidial drugs used in poultry should be considered.

α-Mangostin is a xanthonoid found in different parts of mangosteen (*Garcinia mangostana Linn*.), dominantly in the fruit pericarp. Several articles have reported that the compound is rich in biological activities, including anti-inflammatory, antidiabetic, antioxidant, anticancer, antibacterial, antifungal and antiparasitic activity [10–14]. Due to tremendous benefits, studies on α-mangosteen have been widely explored in various applications. In veterinary medicine, α-mangostin was found to exhibit antiproliferative activity towards spermatogonium and may be utilized as non-surgical castration of male animals [15]. Apart from that, the compound also showed a potent antiproliferative effect on canine osteosarcoma cells by inducing apoptosis cell death [16]. Mangosteen pericarp with or without the combination with ginger rhizome has been reported to enhance broilers’ performance and suppress cholesterol levels of heat-stressed chicken, and the fruit rind powder was reported to enhance the immune system, promote productivity and improve the health benefits of broilers [17,18]. The antiparasitic activity of α-mangostin has been proven in trophozoite and cyst forms of *Acanthamoeba triangularis* in synergistic with chlorhexidine [19]. Currently, several herbal and natural compounds have been investigated as an alternative to conventional anticoccidial drugs. Besides, the use of α-mangostin as a natural antiparasitic agent may be beneficial in replacing chemical antibiotics used at the present due to its promising potential [20,21]. However, α-mangostin is derived from plants that may have high variation in the content and contain several unwanted impurities. The extraction, standardization and precise quantification of the compound are necessary.

This work aimed to standardize α-mangostin content in the mangosteen pericarp extract (MPE), assess the antioxidant activity and investigate the use of MPE formulation as a replacement for anticoccidial drugs in broiler feed. The MPE formulation with a precise and standardized quantity of α-mangostin was obtained and used to add to the broiler feed in place of anticoccidial drugs. To the best of our knowledge, studies covering the extraction and manufacturing of standardized MPE formulation along with in vivo study in broilers have not been reported.

## 2. Materials and methods

### 2.1. Materials

Mangosteen pericarps were obtained from different regions of Thailand. Dichloromethane (DCM), hexane, chloroform, ethyl acetate, acetonitrile, ortho-phosphoric acid, ethanol and methanol were bought from Merck & Co. (Darmstadt, Germany). Ascorbic acid, 2,2-diphenyl-1-picrylhydrazyl (DPPH), 2,2’-azino-bis(3-ethylbenzothiazoline-6-sulphonic acid) (ABTS), potassium persulphate, sodium dihydrogen phosphate dihydrate, 2,4,6-tripyridyl-S-triazine (TPTZ) and galvinoxyl were purchased from Sigma Aldrich (St. Louis, MO, USA). Trypticase soy broth, nutrient broth and agar were acquired from Becton, Dickinson and Company (Sparks, MD, USA). Corn starch, Avicel® PH102 and dibasic calcium phosphate were procured from P.C. Drug Center (Bangkok, Thailand). Other chemicals were used as received.

### 2.2. Sample preparation and identification

#### 2.2.1. Mangosteen pericarp preparation

Mangosteen pericarps were gathered from different regions of Thailand for the standardization and quantification of the active compound (α-mangostin). The pericarps from each source were independently weighed, chopped and sliced, then dried at 55°C until completely dried (the weight of the pericarp is stable). The dried pericarps were then used for the following experiments.

#### 2.2.2. Sample identification

Phytochemical identification of the pericarp samples was performed following Thai Herbal Pharmacopeia (THP) 2020, which included ferric chloride test, vanillin-sulphuric acid test, thin-layer chromatography for identification and other general control methods (heavy metals, total ash, acid-insoluble ash, ethanol-soluble extractive, water-soluble extractive, loss on drying and microbial contamination). The test procedures strictly followed the pharmacopeial directions [22].

The mangosteen pericarps from each source were prepared and identified individually. It was found that the pericarps from all sources passed the identification tests based on the physical characteristics and phytochemical properties of the pericarp. They also passed other quality control tests according to the THP 2020 regulations, including the heavy metal and microbial limits.

### 2.3. Standardization of mangosteen pericarp extract

α-Mangostin, a phytochemical xanthone found in mangosteen pericarp, was extracted using the maceration technique and purified using column chromatography. Briefly, 1 g of the pericarp powder was macerated in 15 mL of dichloromethane (DCM). The mixture was then filtered through a Whatman filter paper No. 1. The filtrate was then purified through a silica gel column (30 cm × 15 mm diameter) using DCM:hexane mixture (4:6) as the eluent. The first 400 mL of the eluent was discarded before the subsequent 500 mL was collected in 5 vials. After that, 15 fractions of the eluent, 50 mL each, were gathered. Then, the solvent ratio of the eluent was altered to 6:4 (DCM:hexane). The purification was monitored by thin-layered chromatography using chloroform:ethyl acetate (85:15) as the mobile phase and detected under UV light at 254 and 366 nm. Common vials were pooled and filtered through a 0.45-μm membrane filter for purification before analysis using high-performance liquid chromatography (HPLC). The calibration curve of the purified α-mangostin was plotted for standardization of the compound in the extract.

### 2.4. High-performance liquid chromatography

α-Mangostin was quantified using an HPLC (Agilent® 1200, Santa Clara, CA, USA) with ReproSil-Pur Basic C18 column (250 mm × 4.6 mm, 5 µm). The mobile phase was acetonitrile (A) and 0.1% ortho-phosphoric acid (B). The sample was eluted using a gradient elution programme as follows: 70% A (0–15 min), 70–75% A (3 min), 75–80% A (1 min), 80%A (6 min) and 80–70% A (1 min). The injection volume was 20 µL, the flow rate was 1.0 mL/min and the detection was performed using a UV detector set at 320 nm [23,24].

### 2.5. Mangosteen pericarp extraction and quantification

The active compound was extracted from the mangosteen pericarp using different solvents and methods, which were modified from Novilla et al. (2016) [25]. One gram of the pericarp was digested in 20 mL of water for 10 min and filtered through a filter paper. Then, the solid was re-digested with fresh water and filtered. The filtrates were pooled, and the solvent was evaporated in a water bath. Furthermore, the same amount of pericarp powder was macerated twice with 6 mL of 50%, 70% or 95% ethanol for 24 h. The MPE was filtered and concentrated under a rotary evaporator. The extracts were diluted with methanol and filtered through a 0.45-μm membrane filter prior to the quantification of α-mangostin using an HPLC.

### 2.6. Antioxidant activity

The antioxidant activity of α-mangostin in the MPE was evaluated using different methods as follows:

#### 2.6.1. DPPH radical scavenging assay

DPPH solution (0.2 mM) was prepared by dissolving DPPH in methanol. Extract sample solution was also prepared in methanol with an α-mangostin concentration of 1 mg/mL. Then, the sample was diluted to various concentrations of α-mangostin ranging between 1 and 1000 μg/mL. DPPH solution was added to each sample, and well mixed before the DPPH-sample mixture was incubated in the dark for 30 min. The scavenging activity was determined using a UV–Visible spectrophotometer at 550 nm. The percentage of radical scavenging activity was calculated using Eq. 2. Ascorbic acid was used as a positive control.

(2) $$\%\;\text{Radical}\;\text{scavenging=1}-\frac{{\text{Absorbance}}_{\text{sample}}}{{\text{Absorbance}}_{\text{control}}}\times \text{100}$$

#### 2.6.2. ABTS assay

A solution of 2,2’-azino-bis(3-ethylbenzothiazoline-6-sulphonic acid) (ABTS) was prepared at a concentration of 7 mM in by dissolving ABTS in a mixture solution of 4.95 mM potassium persulphate and 20 mM sodium dihydrogen phosphate dihydrate (1:1) and incubated in the dark at room temperature for 12–16 h to obtain ABTS^•+^radical. The extract sample was prepared at 1 mg/mL in ethanol, and 100 μL of the sample solution was added to 3.9 mL of the ABTS^•+^radical solution and gently mixed. The absorbance of the mixture solution was measured at 734 nm, and the radical scavenging activity was calculated from the calibration curve of ascorbic acid (positive control) and reported as µg equivalent/mg of the extract.

#### 2.6.3. Ferric reducing antioxidant power (FRAP)

The FRAP reagent was prepared by mixing 10 mM 2,4,6-tripyridyl-S-triazine (TPTZ) with 20 mM ferric chloride solution and 0.3 M acetate buffer pH 3.6 at a volume ratio of 10:1:1. The tests were performed by incubating 180 μL of FRAP reagent with 20 μL of the sample solution (prepared at various concentrations) in the dark for 30 min. The absorbance was measured using a UV–Visible spectrophotometer at 593 nm. Trolox was used as a positive control. The result is reported in Equivalent concentration 1 (EC_1_), referring to the concentration required to reduce Fe^3+^-TPTZ equivalent to 1 mM FeSO_4_•7H_2_O.

#### 2.6.4. Galvinoxyl scavenging study

Galvinoxyl solution (1 mM in methanol, 900 μL) was mixed with the sample solution (1 mg/mL in methanol, 90 μL). The reaction mixture was incubated at 37°C for 20 min, and the absorbance was determined using a spectrophotometer at 420 nm. Ascorbic acid (1 mg/mL) was exploited as a positive control.

### 2.7. Antimicrobial effect

The minimal inhibitory concentration (MIC) of the α-mangostin in the MPE was determined on four *Salmonella* strains using the broth dilution technique. *S. pullorum* and *S. enterilidis* were cultured in nutrient broth, while *S. typhimurium* and *S. gallinarum* were cultured in tryptic soy broth at 37°C. The bacteria were diluted in their respective media to reach the optical density (OD) at 660 nm of 0.08–0.1, providing 10^8^ CFU/mL of the bacteria before being further diluted to 10^6^ CFU/mL. The extract was prepared, and the amount of α-mangostin was quantified prior to the dilution of the samples using twofold serial dilution to obtain 10 concentrations of α-mangostin. The prepared bacterial culture (10 μL) was added to each well before incubation at 37°C for 24 h. The turbidity of each well was observed, and the MIC was recorded.

The minimal bactericidal concentration (MBC) was examined using the spread plate technique. Three aliquots from the first non-turbid wells observed in the MIC experiment were drawn out (100 μL) and spread on trypticase soy agar plates (*S. typhimurium, S. gallinarum*) or nutrient agar plates (*S. pullorum, S. enteritidis*) and incubated for 24 h. The concentration in which the plates had no bacterial growth was recorded as the MBC.

### 2.8. Preparation of mangosteen pericarp extract granules

Concentrated MPE was mixed with various diluents (corn starch, Avicel® PH102 and dibasic calcium phosphate) to make granules of α-mangostin. Briefly, 10 g of the diluent was mixed with the extract at different ratios, as shown in Table 1, to make a damp mass. The mass was spread on a tray and dried at 50°C in a hot air oven. The dried mixture was then sieved through mesh no. 20 to yield the final granules. The optimized product, obtained after evaluation of the formulations, was further up-scaled proportionally with slight adjustments to obtain equivalent granules for broiler feeding.

**Table 1.** The composition of mangosteen pericarp extract formulations.

Formulation	Diluent	Weight of diluent (g)	Weight of extract (g)
F1	Corn starch	10	10
F2	Avicel® PH102	10	10
F3	Avicel® PH102	10	20
F4	Dibasic calcium phosphate	10	10

 The amount of α-mangostin in the granules was quantified using an HPLC following the protocol mentioned above. The granules were accurately weighted, and α-mangostin was extracted with methanol overnight at room temperature. The sample was centrifuged, and the supernatant was collected for quantification of α-mangostin.

### 2.9. Formulation characterizations

The obtained granules were characterized on their physical and chemical characteristics through their appearances, flowability and stability. The appearances of the granules were evaluated visually concerning the granule size, homogeneity, colour and so forth.

#### 2.9.1. Flowability

The flowability of the granules was examined using the angle of repose method. Briefly, a funnel with a wide outlet was affixed at a fixed distance above a white paper. The formulations were weighed (5 g) and filled into a funnel with the outlet closed. Once the outlet was opened, the granules flow formed a cone-shaped pile, and the height and diameter of the pile were recorded. The angle of repose was obtained by calculation according to Eq. 3. The angle of repose obtained was fitted with the Carr index where a value of less than 30° indicates very free flowing, 30–38° is free flowing and 38–45° signifies fair to passable flow [26,27].

$$\theta ={tan}^{-1}\frac{height}{0.5\times \;diameter}$$

#### 2.9.2. Stability

The stability of the formulations was studied under long-term (30 ± 2°C, 75 ± 5% RH) and accelerated conditions (40 ± 2°C, 75 ± 5% RH) following the ASEAN guideline on stability study of drug product. The samples (3 g) were kept in tightly closed clear glass vials under a controlled atmosphere. At the predetermined time points, the vials were taken out for appearance evaluation, and 30 mg of each formulation was sampled for quantification of α-mangostin. The sample was mixed with 1 mL of methanol and extracted overnight before centrifugation at 5,000 rpm for 10 min to collect the supernatant for HPLC analysis.

### 2.10. Preparation of broiler feed

The broilers feed with the ingredients as specified in Table 2 was mixed with the MPE formulation in a high-shear mixer to obtain drug-incorporated feed with α-mangostin equivalent to 50, 250 and 500 mg/kg feed providing the feeds with low, medium and high dose of α-mangostin.

**Table 2.** Ingredients of the broiler feed.

Ingredients	Quantity (g/kg feed)
Ground maize	591.2
Soybean meal	295.2
Corn gluten meal	40.7
Limestone	8.9
Dicalcium phosphate	19.3
Premix	3.0
Choline chloride	1.0
Sodium chloride	3.0
Sodium bicarbonate	1.5
Methionine	2.3
Lysine HCl	2.7
Threonine	0.4
Vegetable oil	30.7

### 2.11. In vivo anticoccidial drug replacement study

#### 2.11.1. Animal

The in vivo experiment in the broilers was approved by the Animal Studies Ethics Committee, Faculty of Pharmacy, Silpakorn University (No. 08/2563). Two hundred and fifty broilers ROSS308 were randomly assigned into 5 treatment groups. All broilers were given ad libitum feeding (without restriction) throughout the experiment. The drugs were withdrawn 1 week before experiment termination (total treating time = 35 days). Each group was fed as follows:

Treatment 1: Control diet

Treatment 2: Control diet with anticoccidial drug (salinomycin 60 mg/kg feed) (positive control)

Treatment 3: Control diet with α-mangostin 50 mg/kg feed

Treatment 4: Control diet with α-mangostin 250 mg/kg feed

Treatment 5: Control diet with α-mangostin 500 mg/kg feed

The animal was fed *ad libitum* twice a day (7.00 am and 4.00 pm) with free access to fresh water throughout the day. At the age of 14 days, *Eimeria tenella* (2,500 oocysts) was orally inoculated to each broiler. The data on body weight, feed intake and mortality of the broilers were recorded on day 0, 21 and 42. Any side effects that occurred during the experiment were recorded to evaluate the safety of the feed containing the extract. Five broilers from each group were randomly selected after the inoculation of *E. tenella* for 7 and 14 days. The caecal appendix of the selected poultry was collected for visual evaluation of coccidial infection, and the lesion score was ranked from 0 to 4 following Johnson and Reid (1970) procedure [28]. Also, the tissue was prepared for histopathology examination according to Luna (1968), where scores from 0 to 4 were given following Korver et al.’s (1997) protocol [29,30]. The data were pooled for the final scoring of the lesion.

#### 2.11.2. Drug residual in broiler’s meat and liver

The meat and liver (n = 3) of the broilers were collected after the experiment and stored at 4°C. The active compound was extracted from the meat by chopping 300 g of the meat and grinding in a meat grinder. Then, 5 mL of ethyl acetate was added to 1 g of the ground meat followed by vortex mixing for 10 min and 10-min ultrasonication. The mixture was then separated by centrifugation at 3,000 rpm for 10 min. The supernatant was collected, and the meat was re-extracted with fresh ethyl acetate. The supernatants were pooled and evaporated under a rotary evaporator. The dried compound was redissolved with 0.5 mL of methanol and filtered through a 0.45-µm cellulose filter prior to the quantification of α-mangostin using an HPLC. For the extraction of the active compound from the liver, 1 mL of distilled water was added to the liver and extracted with 2 mL of hexane twice. The hexane layer was discarded, and the aqueous layer was evaporated under N_2_ flush. The dried compound was redissolved in 0.5 mL of methanol, and the mixture was filtered for HPLC analysis.

### 2.12. Statistical analysis

The data were reported as mean ± standard deviation (SD) for in vitro studies and mean with pooled standard error (SE) for in vivo studies. The statistical analyses were performed using Analysis of Variance (ANOVA) and Duncan’s new multiple range test (DMRT) at a 95% confidence interval. The analyses were carried out using SPSS software.

## 3. Results and discussion

### 3.1. Purification, standardization and content determination

During the purification of α-mangostin in the MPE, the fractions of pure α-mangostin were monitored using TLC compared to the retardation factor of the standard α-mangostin (hRf = 56). Thereafter, the purity of the collected fractions was confirmed using HPLC, where a single sharp peak at an almost exact retention time as the standard α-mangostin was observed, assuring that pure α-mangostin was obtained.

**Figure 1.**
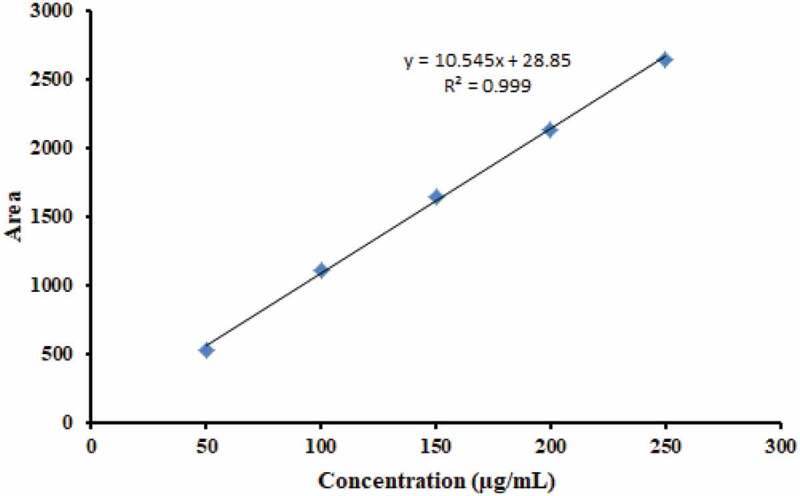
The calibration curve obtained from the extracted α-mangostin.

The α-mangostin in the purified extract was used to generate a calibration curve for content determination, and it was found that α-mangostin concentrations ranging from 50 to 250 µg/mL provided a linear response with an R^2^ of 0.999 (Figure 1). The finding revealed that the use of purified extract could yield a precise and reliable amount of α-mangostin, which can be further used to prepare the standard curve for α-mangostin quantification in the following experiments.

To find the most suitable solvent for the extraction, mangosteen pericarps (pooled from different sources) were extracted with either water, 50% ethanol, 70% ethanol or 95% ethanol. The content of α-mangostin in the extract received from each extraction medium is presented in Table 3. It was found that water was not able to extract α-mangostin from the pericarp, while the most suitable medium was 95% ethanol, which could bring about the highest content of α-mangostin (86.33 ± 0.71 mg/g). Then, α-mangostin from each source was quantified to identify the source of pericarp that provided the highest content of α-mangostin. The highest amount of α-mangostin was 124.75 ± 0.59 mg/g, which was obtained from the mangosteen pericarp received from a farm in the western region of the country. Therefore, the pericarp from this source was selected for all the following experiments.

**Table 3.** The content of α-mangostin extracted from the mangosteen pericarp using different extraction media (*Significant difference from other extraction media (p < 0.05)).

Extraction medium	α-Mangostin (mg/g)
Water	-
50% Ethanol	81.09 ± 0.24
70% Ethanol	83.89 ± 0.32
95% Ethanol	86.33 ± 0.71*

### 3.2. Antioxidant activity

The antioxidant activity of the MPE examined using different methods is presented in Table 4. The DPPH assay determines the amount of DPPH radical scavenged by the MPE into DPPH:H. It was observed that the MPE containing α-mangostin was 3 times more potent than ascorbic acid, which was used as a positive control. In the ABTS assay, the principle is similar to the DPPH assay where the ABTS^·+^ radical is scavenged by the sample. The result was in concordance with the former test, where the tested sample exhibited 3 times greater antioxidant activity compared with the positive control. The FRAP assay determined the concentration of the sample required to reduce Fe^3+^-TPTZ to Fe^2+^-TPTZ compared to 1 mM FeSO_4_•7H_2_O. The result revealed that the pericarp extract was thrice more potent than the positive control, Trolox. Lastly, the sample showed a higher anti-galvinoxyl free radical activity compared with ascorbic acid. All in all, the results from all tests showed that the antioxidant effect of α-mangostin found in the MPE was more potent than that of the known antioxidants (ascorbic acid and Trolox). The oxidant scavenging effects of α-mangostin may be a result from its xanthone structure [31–33]. The structure has been proven to possess several antioxidant mechanisms, including reactive oxygen species quenching, cellular antioxidant defence systems stimulation or prevention of lipid peroxidation [32–34].

**Table 4.** Antioxidant activity of mangosteen pericarp extract quantified using different methods.

Antioxidant experiment (Unit)	Positive control	MPE eq. to 1 mg α-mangostin
DPPH scavenging (%)	Ascorbic acid: 6.16 ± 0.02	19.72 ± 1.28
ABTS (µg equivalent/mg)	Ascorbic acid: 229.74 ± 4.48	796.84 ± 6.29
FRAP (EC_1_)	Trolox: 2728.05 ± 8.98	798.30 ± 3.35
Galvinoxyl (mg/mL)	Ascorbic acid: 19.27 ± 0.99	21.13 ± 0.70

### 3.3. Antimicrobial effect

Prior to the experiment, it was found that the amount of α-mangostin in each diluted sample ranged from 0.33 to 171.39 μg/mL. The MIC of the active compound towards all *Salmonella spp*. strains was 5.36 μg/mL (13.01 μM). However, the MBC varied among the strains. The MBC was equivalent to the MIC (13.01 μM) for *S. pullorum* and *S. typhimurium*. On the other hand, the MBC for *S. enteritidis* and *S. gallinarum* was 10.71 μg/mL (26.02 μM). The findings suggested that the α-mangostin was efficient in killing and inhibiting the growth of common bacteria found in broilers [35]. The antimicrobial effect of α-mangostin on gram-positive and gram-negative bacteria has been reported. In this work, we focused on highly concerned bacteria, which was found to be in concordance with the literature conveying that the compound showed promising antimicrobial effects [36]. Besides, we highlighted the antibacterial effect of α-mangostin against *Salmonella* spp. which is a natural inhabitant of the gastrointestinal tract of birds. The infection of the bacteria in broilers could cause foodborne disease due to the transmission to humans through contaminated meat and derived products [35]. Thus, the infection is highly concerning. The mechanism of action on gram-negative bacteria of the active compound has not been widely discussed; however, reports on gram-positive bacteria showed that α-mangostin targets several metabolic pathways and that the resistance to the compound could be minimal [37,38].

### 3.4. Mangosteen pericarp extract formulation

The dried formulation was obtained with a % yield of 56.98%. The mixture of the MPE and Avicel® PH102 in a weight ratio of 1:1 (F2) was rather too dry and crumbly, but the diluent to extract in a ratio of 1:2 (F3) provided a damp mass that can be mixed and dispersed homogeneously. The % yield of this dried mixture was 55.42%. Lastly, dibasic calcium phosphate was somewhat incompatible with the ethanol in the extract at a weight ratio of 1:1. The % yield of the mixture dropped to 50.01%. The granules of the MPE/Avicel® PH102 mixed at a weight ratio of 1:2 seemed to be the most optimal granule formulation.

#### 3.4.1. Flowability

The flowability results are shown in Table 5. It indicates that upon the formation of extract granules, the flowability of all formulations is impressive. The corn starch formulation had less flowability compared to the other formulations. The flow of Avicel® PH102 and dibasic calcium phosphate formulations was excellent, which in turn be promising candidates for broiler feeding.

**Table 5.** Flowability of each granule formulation performed using the angle of repose technique and fitted into the Carr classification index (n = 3).

Formulation	Angle of repose	Flowability
F1	33.64 ± 2.03°	Free-flowing
F2	-	-
F3	26.45 ± 2.60°	Very free-flowing
F4	27.62 ± 1.68°	Very free-flowing

### 3.5. α-Mangostin quantification and stability

**Figure 2.**
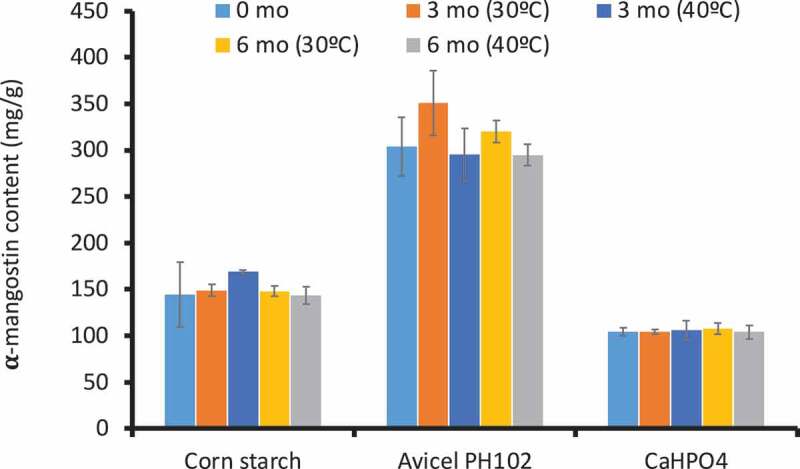
Content of α-mangostin in each formulation at 0, 3 and 6 months under long-term and accelerated conditions.

The quantification of α-mangostin in each formulation is shown in Figure 2. It is predictable that the F3 which was made of Avicel® PH102 and mangosteen pericarp formulation at the weight ratio of 1:2 had the highest amount of α-mangostin (303.89 ± 31.51 mg/g) because the amount of the extract added to this formulation was twice higher than the other formulations. Microcrystalline cellulose was the most appropriate diluent for the preparation of MPE granule formulation in terms of manufacturing suitability, flowability and active compound content.

Upon storage under long-term and accelerated conditions, the appearance of the formulations at all time points was not significantly changed. Corse and flowable granules were still observed. Moreover, the amount of α-mangostin was similar to the pre-storage content (Figure 2). This finding suggested that the formulation and the active compounds can be stored under storage conditions for at least 6 months.

### 3.6. In vivo anticoccidial drug replacement study

After treating the broilers with α-mangostin, it was found the body weight, feed intake, feed conversion ratio and mortality of the boilers in all groups were not significantly different (Table 6) compared with the control group and the group receiving salinomycin (Group 2). The finding was different from Pop et al. (2019), which conveyed that the broilers had reduced weight gain after receiving a different combination of commercial herbal formulas (including *Allium sativum, Thymus serpyllum, Origanum vulgare, Satureja hortensis, Chelidonium majus*, etc.) [21]. However, these formulations also did not significantly improve productive performance of the broilers which is in concordance with our findings and other researchers [39]. This could be due to the differences in the ingredients and quantity given to the broiler. Thus, our results were promising for showing no negative effect on the broiler’s health and welfare. Moreover, the safety of α-mangostin and the extract was satisfying since there was no significant side effect or mortality found in the broilers receiving the extract compared with the control groups. Previous toxicity studies in animals of α-mangostin showed that the compound was safe upon oral administration, where no mortality or sign of toxicity was found when 2000 mg/kg of α-mangostin was given [40].

**Table 6.** Information on broiler feed intake from day 1 to day 35.

	T1	T 2	T3	T4	T5	*P-value*	*Pooled SE*
Initial weight (g)	45.8	45.2	45.5	45.8	45.7	0.484	0.01
Final weight (g)	2614.6	2506.4	2580.8	2642.4	2589.4	0.957	27.55
Weight gain (g)	2568.8	2460.6	2535.0	2596.6	2543.6	0.957	27.55
Feed intake (g)	3727.9	3648.7	3609.2	3777.5	3665.1	0.991	47.14
FCR	1.4	1.5	1.4	1.5	1.4	0.812	0.01
Mortality (%)	0.4	0.2	0.2	0.0	0.0	0.279	0.05

T1: Control diet, T2: Control diet with anticoccidial drug (salinomycin 60 mg/kg feed) (positive control), T3: Control diet with α-mangostin 50 mg/kg feed, T4: Control diet with α-mangostin 250 mg/kg feed, T5: Control diet with α-mangostin 500 mg/kg feed

**Figure 3.**
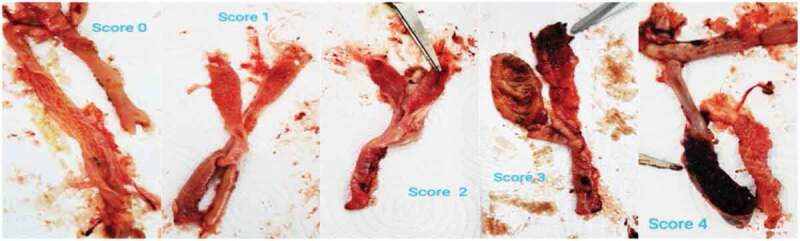
Post-mortem intestinal lesion score from coccidia infection.

Considering the coccidial infection of the broilers after inoculated with *E. tenella* on day 14, the scoring was given according to the appearance of the lesion as shown in Figure 3. The control group had a higher intestinal lesion score post-mortem compared with the broiler receiving salinomycin and all doses of α-mangostin (Table 7). Moreover, the group that received 50 mg/kg α-mangostin showed similar lesion score post-mortem compared with the group receiving the anticoccidial drug. This result suggested that the MPE containing α-mangostin was efficient as a replacement for an anticoccidial drug, even when the α-mangostin was as low as 50 mg/kg feed. In addition, there was no residual active compound in the poultry meat and liver. The result was in concordance with other research studied on plant extract as anticoccidial infection in broilers. Herbal essential oil mixture and garlic extract were previously reported to be used as additional or substituent compounds in coccidiosis broilers [39,41]. Arczewska-Włosek and Świątkiewicz (2013) also reported that alcoholic extract of some plants containing phenolic compounds did not favour anticoccidial activity; such herbs consist of rosemary, sage, oregano, nettle, purple cone-flower and thyme. Interestingly, garlic also did not show anticoccidial effect although 750 mg of the extract/kg feed was given [42]. Many articles have confirmed that α-mangostin in the mangosteen pericarp contains excellent biological activity, including anticoccidial activity [10,43,44]. Previous studies conveyed that the phenolic compounds in the food component could effectively control parasitic diseases and the use of the compound is a cost-effective method to improve the health of livestock and poultry against parasitic diseases [19,45,46]. The MPE contains α-mangostin (a polyphenolic xanthonoid), flavonoids and other phenolic compounds, which are rich in biological activities [47]. Pomegranate peel extract, which contains similar phytochemicals to MPE, has also been proven to be beneficial for anticoccidial effect. It is reported that the extract, in a dose-dependent manner, reduced the intestinal lesion and decreased the excretion of *Eimeria* oocytes in the faeces. However, a higher dose of the extract has negative effects on the poultries’ liver tissue, feed intake and weight gain [48]. In contrast, the purified compound of α-mangostin in the present work showed no negative effect on the chicken and acted as a natural anticoccidial compound similar to a chemical agent. However, the mechanism of action of α-mangostin towards *Eimeria* spp. has not been precisely reported and could be established in the future.

**Table 7.** Coccoidal lesion score of the broilers (post-mortem) with different interventions.

	T1	T2	T3	T4	T5	*P-value*	Pooled SE
Lesion score	2.1^a^	1.4^b^	1.3^b^	1.1^b^	1.5^b^	0.042	0.07

^a,b^Means within a row with no common superscript differ significantly (P < 0.05). T1: Control diet, T2: Control diet with anticoccidial drug (salinomycin 60 mg/kg feed) (positive control), T3: Control diet with α-mangostin 50 mg/kg feed, T4: Control diet with α-mangostin 250 mg/kg feed, T5: Control diet with α-mangostin 500 mg/kg feed

## 4. Conclusion

The mangosteen pericarp collected from different local sources was found to contain α-mangostin in a satisfactory quantity. The extraction and purification methods performed were able to provide purified α-mangostin. The extracted compound was standardized and able to create a linear calibration curve. The compound had excellent antioxidant activities and antimicrobial activity towards *Salmonella spp*. The extract was formulated into granules; using Avicel® PH102 as the diluent was the most promising, offering pleasant flowability, high α-mangostin content and good stability. In a mixture of broiler feed, α-mangostin was safe for the broilers and could replace the use of antibiotic (salinomycin) 60 mg/kg feed once given at only 50 mg/kg feed. Importantly, the active compound in the extract did not affect the growth rate of the broilers and did not deposit in the meat or liver of the broilers. All in all, the MPE presented to be a natural antioxidant and natural antibiotic which could be used to replace the anticoccidial drug additive in broiler feed.
